# Alignment of roles of near-peer mentors for medical students underrepresented in medicine with medical education competencies: a qualitative study

**DOI:** 10.1186/s12909-019-1854-x

**Published:** 2019-11-11

**Authors:** Amy Prunuske, BreAnna Houss, Anna Wirta Kosobuski

**Affiliations:** 1 Medical College of Wisconsin- Central Wisconsin, 333 Pine Ridge Blvd. Suite 2-730, Wausau, WI 54401 USA; 2 University of Minnesota Medical School Duluth Campus, 1035 University Dr, Duluth, MN 55812 USA

**Keywords:** Peer mentors, Prematriculation, Underrepresented in medicine, Medical students, ACGME competencies, Near-peer

## Abstract

**Background:**

Medical student learning experiences should facilitate progressive development of competencies required for practice. Medical school training opportunities have traditionally focused on acquiring medical knowledge and patient care competencies while affording less opportunity to receive feedback on practice-based improvement and system-based practice competencies. The Prematriculation program at the University of Minnesota Medical School Duluth Campus (UM MSD) utilized near-peer mentors to support the transition of students underrepresented in medicine, including American Indian/ Alaska Natives (AI/AN) and those from rural backgrounds, into medical school. The purpose of this study is to better define the role of near-peer mentors and explore the alignment of near-peer mentorship with the ACGME core competencies.

**Methods:**

An important component of the Prematriculation program, designed to prepare incoming under-represented students for medical school, was the inclusion of near-peer mentors. The six near-peer mentors participated in semi-structured interviews or focus groups within 1 year of serving as a near-peer mentor. Themes emerged from open-coding of the transcripts.

**Results:**

The near-peer mentors drew on their own experiences to transmit information that supported the socialization of the matriculating students into medical school. Direct benefits to the mentors included solidifying their own understanding of medical knowledge and execution of procedural skills. Mentors provided examples of benefits related to their own development of interpersonal communication and professionalism skills. Operating in the context of the program provided opportunities to engage mentors in practice-based improvement and system-based practice.

**Conclusions:**

Serving as a near-peer mentor offers significant benefits to medical students from backgrounds underrepresented in medicine. By taking on the peer mentoring leadership role, students progressed toward the competencies required of an effective physician. Given the importance of acquiring these competencies, it is worth considering how near-peer mentoring can be applied more broadly across the medical school curriculum.

## Background

A major purpose of undergraduate medical education is to prepare students for their future duties as physicians. Several recent competency initiatives have tried to link trainee learning outcomes with the needs of patients [1], as such, the Accreditation Council for Graduate Medical Education (ACGME) in the United States has adopted the following six core competencies [2]:

- Patient Care and Procedural Skills
- Medical Knowledge
- Interpersonal and Communication Skills
- Practice-Based Learning and Improvement
- Professionalism
- Systems-Based Practice

Practice-based learning, working in teams, and systems-based practices are required in evolving health care delivery systems and undergraduate medical education needs to incorporate relevant learning experiences and assessments to help students achieve these competencies [3]. Experiential learning and leadership opportunities facilitate development in these areas and can occur in non-clinical settings. For example, students serving as near-peer mentors to more junior medical students may present important learning opportunities to develop these competencies.

Near-peer mentoring creates occasions for growth of mentees and mentors alike [4]. Peer mentors are at comparable stages as mentees, as compared to near-peer mentors (NPMs), who are slightly more advanced [5]. NPM relationships may be particularly beneficial for students identifying with groups underrepresented in medicine who may feel isolated, lack confidence, and experience higher rates of cultural dissonance [6]. Faculty may benefit from collaborating with underrepresented medical students who bring diverse forms of social and cultural capital into program development and evaluation [7].

Near-peer mentoring occurs in various forms in medical schools, but has not been well-studied [8]. Pre-clinical medical students serve as mentors to high school students as part of pipeline programs [9] or to orient matriculating students to the local environment. As part of the formal curriculum, NPMs serve as tutors for basic science coursework [10]. Mentorship is generally associated with professionalism characteristics such as leadership, responsibility, and guidance. Interaction with mentees can further develop communication skills including active listening and effective transmission of information and expectation, which can be applied towards subsequent professional development and success. NPMs act as advisors, coaches, and mentors for junior students; however, successful relationships hinge on clarity and consensus of roles [11].

The aim of this study was to explore how serving as an NPM for a Prematriculation program can help 2nd year medical students begin to develop the skills necessary to be successful physicians. The Prematriculation program students and their NPMs were American Indian and Alaskan Native (AI/AN) and rural students, who have been historically underrepresented in medicine. In this study, we sought to better define the role of a NPM and explore the alignment of near-peer mentorship with the ACGME core competencies.

## Methods

### Institutional context

The University of Minnesota Medical School Duluth Campus (UM MSD) is a regional campus with a social mission to train physicians who will practice family medicine in rural, Minnesota and/or AI/AN communities and was founded in 1972. UM MSD is a national leader in these areas with a match rate of 40% of graduates in family medicine and over 100 Native American physicians trained [12]. During the time of this study, 60 students were admitted each year to UM MSD, attending basic science courses for 2 years in Duluth and then completing their clinical training at the University of Minnesota Medical School Twin Cities campus.

### Prematriculation program

To support the transition of underrepresented students into medical school, the UM MSD Center of American Indian and Minority Health ran a Prematriculation program from 2013 to 2017 [13] for students that had successfully completed their undergraduate baccalaureate degrees and been accepted to medical school. The goal of the 4-week in-person course was to build the students’ self-efficacy and promote academic success. As part of the interdisciplinary course, there was an infectious disease theme each week; for example, tuberculosis, around which students engaged in learning activities and were assessed with multiple- choice examinations. Weekly learning activities included a problem-based learning case, faculty lectures, clinical skill session, oral presentations on similar cases, and microbiology laboratories. Participants met the underrepresented in medicine criteria set forward by the granting agency Department of Health and Human Services Health Resources and Services Administration (HRSA) [14] and supplemental funds were provided by the UM MSD Dean’s Office to support the inclusion of students who were deemed academically at-risk (undergraduate science GPA below 3.6 and/or MCAT below 27) and had a high interest in practicing rural, family medicine.

Nine NPMs were associated with the program and were selected by the course faculty from the previous year’s class based on exceptional interpersonal skills and strong academic performance during the Prematriculation course and their first year of medical school. For each cohort, there were 1–3 mentors and an average of 8 matriculating students, resulting in a ratio of 1 NPM for every 4.5 participants (Table 1). The demographics of the NPMs were 67% female, 78% from out-of-state, and 78% underrepresented minority with most of the underrepresented minorities being AI/AN. These matched the demographics of the Prematriculation program students, but are meaningfully different from the entire matriculating class, which included only 11% underrepresented minority and 13% from out-of-state.

**Table 1:** Demographics of Prematriculation Program Mentors and Students

Year	Number of Mentees	Number of Mentors	Ratio Mentees:Mentors	Female mentor(s)	Under-representedMinority mentor(s)	Out-of-state mentor(s)
2013	6	1	6	1 (100%)	1 (100%)	1 (100%)
2014	3	1	3	1 (100%)	1 (100%)	1 (100%)
2015	13	3	4.3	2 (66%)	2 (66%)	2 (66%)
2016	9	2	4.5	1 (50%)	1 (50%)	1 (50%)
2017	9	2	4.5	1 (50%)	2 (100%)	2 (100%)
Average	8	1.8	4.5	1.2 (67%)	1.4 (78%)	1.4 (78%)

The Prematriculation course occurred during the summer when the NPMs had no other academic commitments. NPMs were paid an hourly salary and were expected to attend the Prematriculation course sessions, support Prematriculation students, lead discussion sessions to prepare for the examinations, and write exam questions. They assisted course faculty with program planning, making quality improvement suggestions and envisioning new learning experiences. During the summer, NPMs met regularly with the course faculty to discuss student challenges, provide feedback, and suggest course improvements.

### Qualitative methods

Multiple measures were taken to ensure trustworthiness, validity, and rigor in the qualitative data analysis [15–17]. NPMs were interviewed 6 months after the end of the Prematriculation program, which was midway through their second year in medical school, by a trained qualitative research assistant not involved in the medical school curriculum or the Prematriculation program. In years when there was more than one NPM, focus groups were conducted to allow the mentors to build on each other’s opinions and experiences. All 6 NPMs from 2014 to 2016 were invited and chose to participate in an in-person interview or focus group to discuss their experience. The course faculty developed the focus group and interview script found in Additional file 1. The interviews were held in the medical school and the duration of the discussion was 60 min. NPM participants were provided a meal and sessions were digitally recorded and transcribed with the removal of any identifying information.

The theoretic framework included a grounded approach to generate ideas about NPM from the data followed by a thematic content analysis [15]. The transcripts were reviewed independently by the course director (AWK) and a faculty member (AP) each year and by the third year, saturation of themes had been achieved. The research team comprised of AWK, AP, and a medical student (BH) immersed ourselves in the data to create and revise codes [17]. BH was a previous program participant and NPM in the program the year after saturation was achieved; this distinct background brought with it the ability to provide additional student insight and rigor to the coding. Transcripts were repeatedly read and codes were revised and clustered by team members after discussion. Code tables were generated and the team consulted the literature to generate themes and explanatory models. NPM codes were cross-checked with codes previously identified from the program participants [13]. The transcripts were subsequently reviewed by the coding team to ensure that codes matched and accurately reflected the intent of the participants’ responses. To ensure privacy, NPMs were randomly assigned identities (indicated by NPM1, NPM2...) to differentiate individual comments. As a final step, the formulated theories were compared to existing frameworks including the ACGME competencies.

## Results

### The role of the near-peer mentor

We sought to use feedback from the near-peer mentors to clarify their role in the Prematriculation program. There is a continuum of guidance including mentors, coaches, and advisors, encountered in medical school. An advisor might use words like “you should”, a coach might ask “What could you do?”, and a mentor shares “in my experience, this has worked.” [18] Based on our interviews, NPMs indicated they offered value as mentors drawing on their previous experience and insight into the students’ concerns in contrast to course faculty who were described as serving in more of an advisory role with greater understanding of the school and disciplinary-based knowledge (Table 2).

**Table 2:** Contrasting Roles for Near-Peer Mentors and Faculty Advisors in Prematriculation Program

	Near-peer mentor	Faculty advisor
Focus	Socialization into medical school. Led discussions with the learners to transmit information based on own recent experience and provide emotional support	Taught specific sessions during the course and played organizational and evaluative role
Expert	In experience and expectations of a first year medical student	In disciplines and medical education
Types of interactions	Repeated informal interchanges including beyond program to discuss study strategies and activities outside of school	Conducted course sessions with targeted learning objectives and activities
Illustrative Quote	“Helps the students along and makes them feel supported, like they can contact me anytime if they need anything” (NPM 4)	“Some of the faculty were like PhDs or have never gone to medical school, or they went to medical school but it was like 20 or 30 years ago.” (NPM 4)

The NPMs described their role as being beneficial to the Prematriculation participants in a number of important ways; these included 1) emotional/personal support; 2) academic support; and 3) ‘insider view’ of medical school experience. Only a year prior, the NPMs had been in the shoes of the new students: processing the realization of entering medical school, grappling with adaptation to a new location, lifestyle and medical school environment while asking themselves if they could succeed. Having lived the experience, the NPMs empathized with the new students, (“you remember what it feels like to go through that and to make that transition” (NPM 6) and were able to translate this to strategies to meet the participants’ needs, i.e., make school “seem less intimidating...that was my goal, to make it seem like it’s doable” (NPM 2). NPMs noted that the small cohorts and opportunities for frequent interaction strengthened the mentoring relationship. Informal mentoring was emphasized including social opportunities and continuing support during the first year of medical school. Thus, a critical role of the NPMs was to provide engaged empathic support sensitive to the unique needs of the program participants; similar sentiments were emphasized previously from focus groups with the program participants [13].

### Core competencies

In addition to the benefits the NPMs provided to the matriculating students, the NPMs found the experience to be beneficial to their own skill development. The NPMs found that the program helped them to consolidate their patient care and medical knowledge, reinforced their commitment to rural and AI/AN health, and contributed to communication and professionalism skills. These themes highlight the development of the ACGME competencies (Table 3).

**Table 3:** NPM Roles and Prematriculation Program Examples by ACGME Competency

Competency	Definition	Near-peer mentor roles
Patient Care and Procedural Skills	Provide patient care that is compassionate, appropriate, and effective for the treatment of health problems and the promotion of health.	Teach and supervise clinical skills. Assist near-peers with transitions in a compassionate manner.
Medical Knowledge	Demonstrate knowledge about established and evolving biomedical, clinical, and cognate (e.g. epidemiological and social-behavioral) sciences and the application of this knowledge to patient care.	Explain, organize, and assess medical knowledge. Share evidence-based resources
Practice-Based Learning and Improvement	Investigate and evaluate their patient care practices, appraise and assimilate scientific evidence, and improve their patient care practices.	Reflect and improve on own training experiences. Review outcome data and participate in scholarly activities.
Interpersonal and Communication Skills	Demonstrate interpersonal and communication skills that result in effective information exchange and teaming with patients, patients’ families, and professional associates.	Demonstrate effective communication with program participants, co- near peer mentors, and course faculty.
Professionalism	Demonstrate a commitment to carrying out professional responsibilities, adherence to ethical principles, and sensitivity to a diverse patient population.	Demonstrate professional conduct and accountability Positive response to constructive criticism.
Systems-Based Practice	Demonstrate an awareness of and responsiveness to the larger context and system of health care and the ability to effectively call on system resources to provide care that is of optimal value.	Understand institutional mission and its relationship to the community’s health care needs. Utilize system resources and work in teams to advocate for optimal outcomes.

The definition column includes description from the 1999 approved language for the ACGME Competencies [19]. The roles column describes knowledge, skills, and attitudes tied to a competency that can relate to near-peer mentors

### Patient care and procedural skills

Patient care is the foundation of medicine and NPMs will often be expected in their careers to be empathetic educators. Broader trends in medical education have included incorporating more clinical exposure earlier in the curriculum [20]. The initial Prematriculation curriculum had no hands-on clinical teaching sessions, but did include clinically oriented problem-based learning cases. This changed after the third year when the NPMs wanted to develop sessions on history taking and basic procedural skills, like suturing, to give the matriculants early exposure to clinical skills. The NPMs benefited by reviewing their own learning and by serving in a supervisory role, which is an important component of the patient care competency. NPM 6 noted:

“… with doing more skills sessions; I think that was a big thing. I know when we did the suturing session, … I had only sutured once before so I walked into this and, I’m like, we’re teaching this now … like, what have you got … because you have to teach them. … So that’s something that you think back on and we try and reflect on what we thought was important from the skills.”

The Prematriculation program offered a low-stakes environment to begin this role modeling since additional training would be provided to the matriculating students before these skills would be performed on patients.

The NPMs strived to bring compassion to the participants’ transition since they desired “to ease that uncomfortableness that some students have” (NPM 6).” The NPMs also have a valuable role to play in promoting self-care in medical school and sharing with the students beneficial study and coping strategies, e.g., emphasizing the need for enough sleep and finding opportunities to destress. Patient care is the foundation of medicine and mentors can be key to building the clinical skills and empathy necessary to be successful in medicine.

### Medical knowledge

NPMs are often ideally suited to aid in educating junior students in medical knowledge being acutely aware of how they struggled to learn the material. A major theme identified from the NPMs was that serving in the teacher role enabled them to consolidate their medical knowledge. The NPMs ran review sessions and wrote clinically-relevant exam questions. “When you teach it, when you create questions, you realize what the high-yield stuff is, what is testable. It helps you develop those skills, which helped me now studying for tests.” (NPM 3). The program helped both the NPMs and participants to better understand the material and to think through its clinical applicability. The NPMs suggested study activities to “make sure they understand it and not just memorize things and not knowing what it is.” (NPM 4).

The NPMs also felt an important responsibility in helping the participants to identify internal and external resources to access reliable information. “It’s nice having somebody that has already been through it that is able to tell you, like, okay this is what these people look for; this is where you can do this, this, and this.” (NPM 5). NPMs’ enthusiasm and organizational experience assisted in the assimilation and application of the large volume of medical knowledge encountered in medical school.

### Practice-based learning and improvement

The NPMs for the Prematriculation program were afforded a valuable opportunity to engage in self-reflection and quality improvement around medical education following their first year of medical school. “Things just like stepping up into the leadership role, is different than being a student sitting through it again. You kind of had more experience and you get to reflect more on your experience of when we did in the Pre-Mat course because it’s kind of like they’re going through, you reflect more to give them advice.” (NPM 6). The NPMs self-directed their own goals for the summer, identifying sessions and materials they wanted to teach the program participants and worked with the faculty to identify evidence-based resources.

One of the NPMs became part of the research team, which provided insight into the value of the mentoring experience. This NPM reviewed program outcomes, appraised medical education literature, and assisted with the analysis and interpretation of the transcripts. Being involved with the Prematriculation program raised her awareness of the medical competencies, but by going a step further and becoming involved with the analysis of the peer mentor program, she solidified her understanding of these competencies and further developed her ability to communicate her and her peers’ experiences. She also felt inspired by the hope that these findings might contribute to advocacy for and empowerment of students at other institutions who are underrepresented in medicine. The Prematriculation program was a unique opportunity for the NPMs to engage in practice-based improvement reflecting on their own experience in the Prematriculation program and contributing student perspective into the evaluation of the program.

### Interpersonal and communication skills

In the context of the Prematriculation program, NPMs needed to be able to demonstrate effective communication with program participants, course faculty, and co-NPMs. The NPMs occasionally had personal commitments that required them to rely on and coordinate coverage of program duties with their co-NPMs and the faculty. NPMs valued feeling part of a team and “being able to collaborate with some of the professors” (NPM5) and learning to recognize when they needed to initiate discussions or share information with other team members.

The NPMs needed to establish trust with the Prematriculation students, to be open to hearing their needs, and to act as a bridge to facilitate communication between the students and the faculty.

“I knew like as a student sometimes it’s scary to ask a professor, so I had a few times where the students would ask me the question like: “Why did I get this wrong?” And I would try to explain it to them, and if I couldn’t, then we could bring it to the professor. That kind of helped ease that uncomfortableness that some students have about just approaching a professor” (NPM6).

The students needed to maintain an open dialogue using nonverbal and verbal techniques, similar to those that they will use with patients, to work with the participants to clarify their understanding of the situation. The students were often from very different backgrounds and the NPMs needed to understand their unique challenges and make sure the students felt welcome.

### Professionalism

The NPM position required fast and consistent turnaround on task completion such as compilation of feedback for each student, organizing weekly programmatic activities, and preparing for clinical training instruction. Management of this workload called for NPMs who were responsible and committed, as noted by the NPMs themselves the role required someone who is “reliable” (NPM 4), “is driven to be there for the students” (NPM 5) and “motivated to help the students learn” (NPM 6). Professionalism also calls for placing the needs of a diverse set of others above self, “it felt really good to just be there and help them out … I just wanted to make it as easy and seamless as possible transition for them” (NPM 6). Being a peer-mentor specifically and coming back to assist with the program came from a sense of responsibility to give back to the program.

The NPMs also recognized the responsibility involved in learning to humanely respond to criticism from participants and using feedback as an opportunity for growth.

“So there was little things like that that you kind of just accepted being a leader, and professors are being criticized all the time and they kind of just have to deal with it so you learn little things like that when you’re actually placed in front of the students.” (NPM 6).

Finally, NPMs needed to maintain participant privacy and confidentiality while also being self-aware of their own limitations. The NPMs successfully upheld this expectation. The program faculty observed this and agreed that this experience supported NPMs in their professional development as future physicians when they will be accountable not only to patients but also to their peers and colleagues.

### Systems-based practice

Medical students should have an understanding of how medical school outcomes relate to the community’s health care needs. Serving as a NPM to other students interested in serving rural and AI/AN communities, helped to reinforce the students’ commitment to addressing these needs.

“It was fun to be with a bunch of students who share a common interest in Native health or their Native identity. And, it reinforced my desire to go into medicine, work amongst Native populations, and it’s just neat to connect with them, even as a mentor or as a peer.” (NPM3).

Another component of system-based thinking is being able to effectively call on system resources. NPMs felt their insider knowledge helped them to advocate for the participants’ needs. “I made a recommendation last year to have the exams or quizzes on Fridays because in Foundations you have them on Fridays.” (NPM 4). Discussions with the course faculty allowed for NPM engagement in system-based thinking and brought the perspectives of students underrepresented in medicine into the system when discussing challenges, adding valuable feedback into the system. The NPMs were able to learn more about the larger system of medical education while supporting the success of students that have been traditionally underrepresented.

## Discussion

NPMs draw on their own recent experiences to provide guidance to support peer success. As this study suggests, the notion of employing near-peer mentorship as a framework toward proficiency in the ACGME competencies places a compelling spin on the potential uses of NPMs. The positive outcomes for the mentees [13] and as shown here mentors, all of whom were from groups underrepresented in medicine, suggests double the impact. Even though direct patient care was absent from the program, we can still view the program experiences as a catalyst for development of physician professional competencies.

The Prematriculation program model presented here provided a structure that enabled NPMs to move through a process that nurtured skills needed for future mastery of professional competencies. As with a patient experiencing unfamiliar symptoms we previously discussed a number of uncertainties and anxieties experienced by incoming medical students, the mentees of the NPMs [13]. This qualitative research study revealed that, in response, the NPMs had moved through a process of assessing, formulating and implementing a course of action. NPMs also had to provide ongoing follow up to ensure the maintenance and alleviation of those concerns. Employing a grounded theory approach revealed key practical insights relevant to the experience of NPMs, such as importance of interaction skills, empathy, and personal insight, utilizing program and institutional resources, and excellence in collaboration and teamwork with other NPMs and faculty. Additionally, the interview and focus group data illustrate NPMs’ understanding and reflection as to what they, as peer mentors, had the capacity to handle and when it needed to be referred out to those with different level of expertise, the faculty. The outcome of this process then moves the mentor through stages much like Miller’s Pyramid [21], from one who knows and knows how as a first year student, to one shows as a NPM and has begun to successfully progress to one who does.

There could be a concern that the NPMs might emphasize less than ideal habits in the matriculating students, but this was not our experience. Based on faculty observation, the NPMs showed consistent maturity in their advice and growing awareness of their own limitations. Others have also reported that NPMs are as effective and are often better received than more formal tutors [22]. NPMs did not receive formal training for their roles having been previous participants in the program and their quality performance did not warrant it. The faculty worked closely with the NPMs and met weekly to provide feedback to support mentor self-efficacy.

The NPMs indicated that receiving formalized written feedback would have been useful toward their development and this study helps to identify the competencies and activities that can be used for providing this feedback. A next step in building the 6 competencies into mentoring experiences would be to give near-peer mentors individualized feedback that incorporates direct observation, participant feedback, and opportunities for individual goal setting to build in self-directed learning [23]. The competencies should be discussed with trainees since they are an important part of residency and students would benefit from training that allows them and colleagues to actively monitor their development.

A study limitation is that the NPMs were from a single institution with a specific mission to train students from groups underrepresented in medicine. It is important to recognize; however, that the small cohort of underrepresented students here are AI/AN. As such, they are in reality an overrepresentation of AI/AN medical students who represent only 0.22% of medical students nationwide making the group a substantial cohort [24]. Nonetheless, the research team also acknowledges that having standardized metrics and milestones on near-peer mentoring would facilitate cross-institutional research and enhance generalizability. The implications of our research are important for supporting the success of students from groups underrepresented in medicine and research from other fields supports the ideas that NPMs directly contribute to the ability of students to persist through academic adversity [25].

## Conclusions

We strongly encourage schools to consider the development of near-peer mentoring opportunities. A robust experience will facilitate opportunities to initiate building foundational skills and development in the 6 competencies by promoting self-efficacy. Providing medical students with these types of experiences is paramount to easing transitions in medical school for students underrepresented in medicine. Furthermore, teaching and providing feedback are underappreciated skills that are an increasing expectation for residents that the near-peer mentoring experience helps to develop.

## Supplementary information

**Additional file 1.** Peer Mentor Interview Script

## Data Availability

To ensure participant privacy, project data is not available publicly.

## Abbreviations

ACGME: Accreditation Council for Graduate Medical Education
AI/AN: American Indian/Alaska Native
GPA: Grade Point Average
HRSA: Health and Human Services Health Resources and Services Administration
IRB: Institutional Review Board
MCAT: Medical College Admission Test
NPM: Near-peer mentor
UM MSD: University of Minnesota Medical School Duluth Campus
UM MSTC: University of Minnesota Medical School Twin Cities
