# Supplementary material for: Alignment of roles of near-peer mentors for medical students underrepresented in medicine with medical education competencies: a qualitative study
Source: BMC Med Educ. 2019 Nov 11;19:417. doi: 10.1186/s12909-019-1854-x (PMC6849195; doi:10.1186/s12909-019-1854-x)
Supplement: Supplementary file 1 — Additional file 1. Peer Mentor Interview Script [file 12909_2019_1854_MOESM1_ESM.docx]

**Peer Mentor Interview Script**

*Thank you for participating in an interview about the Center of American Indian and Minority Health Pre-Matriculation Program. My name is XXX and I am currently working with the Center to analyze this year’s program and plan for improvements towards next year’s program. Our goal for this interview is to learn about your role as a peer mentor in the Pre-Mat Program and gain any insight that might lead towards improvements of the program. Your participation in this interview is completely voluntary and your decision to participate or not will not affect your current or future relations with the University. If you have decided to participate, you are free to withdraw at any time without affecting those relationships. While we are audio recording our conversation today, your responses will remain anonymous.*

*Okay, let’s begin.*

*Section One*

1. *What was your understanding of your responsibilities within the program?*
2. *How did serving as a Peer Mentor help you to develop your skills further, including study skills, knowledge, teaching skills, etc.? How do these skills differ as a mentor from the skills you developed as a participant of the program?*
3. *What are your thoughts regarding the curricular component you developed such as the clinical skills?*
4. *What did you personally and professionally take from being a mentor of the program?*
5. *What sort of support did you receive? Personal, professional, compensation, etc.? And, was it adequate?*
6. *Would it have been helpful to have had training in teaching or leadership prior to taking on this role?*
7. *Would you have liked to have gotten feedback on your teaching from the participants or the faculty?*
8. *In the future, would you recommend having a single peer mentor or a team of peer mentors?*
9. *As a mentor, how did you contribute to easing the transition of students into medical school?*
10. *What sort of academic guidance did you provide for the students?*
11. *How did serving as a peer mentor help to develop the students’ study skills?*
12. *What are some specific programmatic functions that helped the students?*
13. *What are your opinions of the programs structure and organization?*
14. *What were some differences between last year and this year, and how did these differences affect the program/students’ experiences?*
15. *How often do you interact with the peer group of Pre-Matriculation students since the school year began?*
16. *Do you feel like four weeks is an appropriate amount of time for the program?*
17. *What type of person would be good as a peer mentor?*
18. *What would be your recommendations for improvements of the program?*
19. *Anything else you would like to add about anything?*

*Thank you so much for your participation.*
